# Fecal microbiota transplantation-current perspective on human health

**DOI:** 10.3389/fmed.2025.1523870

**Published:** 2025-03-14

**Authors:** Zixuan Cao, Tingting Gao, Ousman Bajinka, Yali Zhang, Xingxing Yuan

**Affiliations:** ^1^Heilongjiang Academy of Traditional Chinese Medicine, Harbin, China; ^2^Country School of Medicine and Allied Health Sciences, University of The Gambia, Banjul, Gambia

**Keywords:** fecal microbiota transplantation, human health, metabolic disorders, microbiome medicine, gut microbiota

## Abstract

Recently, microbiome medicine has attracted the attention of researchers. While this rapidly growing medical approach for various diseases and disorders is changing the paradigm, it is imperative to weigh both its benefits and the associated risk factors. For instance, manipulation of the gut microbiota (GM) has positive effects on metabolic and neurodegenerative diseases. Notably, fecal microbiota transplantation (FMT), a complex method, has shown promise; however, many doubt its feasibility without adverse effects on human health. Given the number of human clinical trials investigating FMT for the treatment of various disorders, this review summarizes recent findings on its impact on human health. This review summarizes the metabolic responses associated with FMT and their reversal effects on gastrointestinal infections, behavioral changes, and immune responses. Additionally, this review discusses the role of FMT in antimicrobial resistance and its co-supplementation effects on human health, safety, potential risks, limitations, prospects, and recommendations. Although this review does not cover all the studies in the database, the searched terms for FMT and human health in clinical trials are sufficient to provide a summary of the current perspective.

## 1 Introduction

Fecal microbiota transplantation (FMT) is a therapeutic intervention that involves transferring processed stool from a healthy donor to restore the balance of gut microbiota (GM). Despite the complexity of microbial taxa and their interactions with the human immune system, FMT has emerged as a promising standard of care (1). Unlike dietary interventions that gradually enrich short-chain fatty acid (SCFAs)-producing taxa, FMT enables the rapid restructuring of gut microbial communities through direct microbial transfer. This approach has shown systemic benefits ranging from metabolic regulation (e.g., blood pressure and glucose homeostasis) to suppression of pathogenic bacterial proliferation (2).

In the management of infectious disease, FMT outperforms first-line therapies for recurrent *Clostridioides difficile* infection (CDI), achieving superior sustained remission rates compared to vancomycin therapy (3, 4). Single-dose FMT administration following antibiotic treatment significantly reduces CDI recurrence and mitigates the transfer of antibiotic resistance genes (5, 6). Beyond its established role in CDI, FMT demonstrates therapeutic potential across multiple disease domains. In virological applications, FMT has facilitated hepatitis B e antigen clearance and addressed human immunodeficiency virus-associated dysbiosis (7, 8). In oncology, FMT modulates the GM to reprogram tumor microenvironments, particularly enhancing anti-programmed cell death protein 1 therapy responsiveness in melanoma treatment (9, 10). Neurological benefits emerge through FMT’s capacity to improve both motor and non-motor symptoms in Parkinson’s disease while enhancing gut microbial diversity (11, 12). Metabolic improvements are achieved via microbial restructuring, including triglyceride reduction and inflammatory pathway regulation (13). Furthermore, postoperative outcomes further highlight FMT’s clinical value, with reduced hospitalization duration, accelerated bowel function recovery, and improved nutritional markers (14). This multifaceted therapeutic profile positions FMT as a versatile intervention across distinct pathophysiological mechanisms.

**Figure 1:**
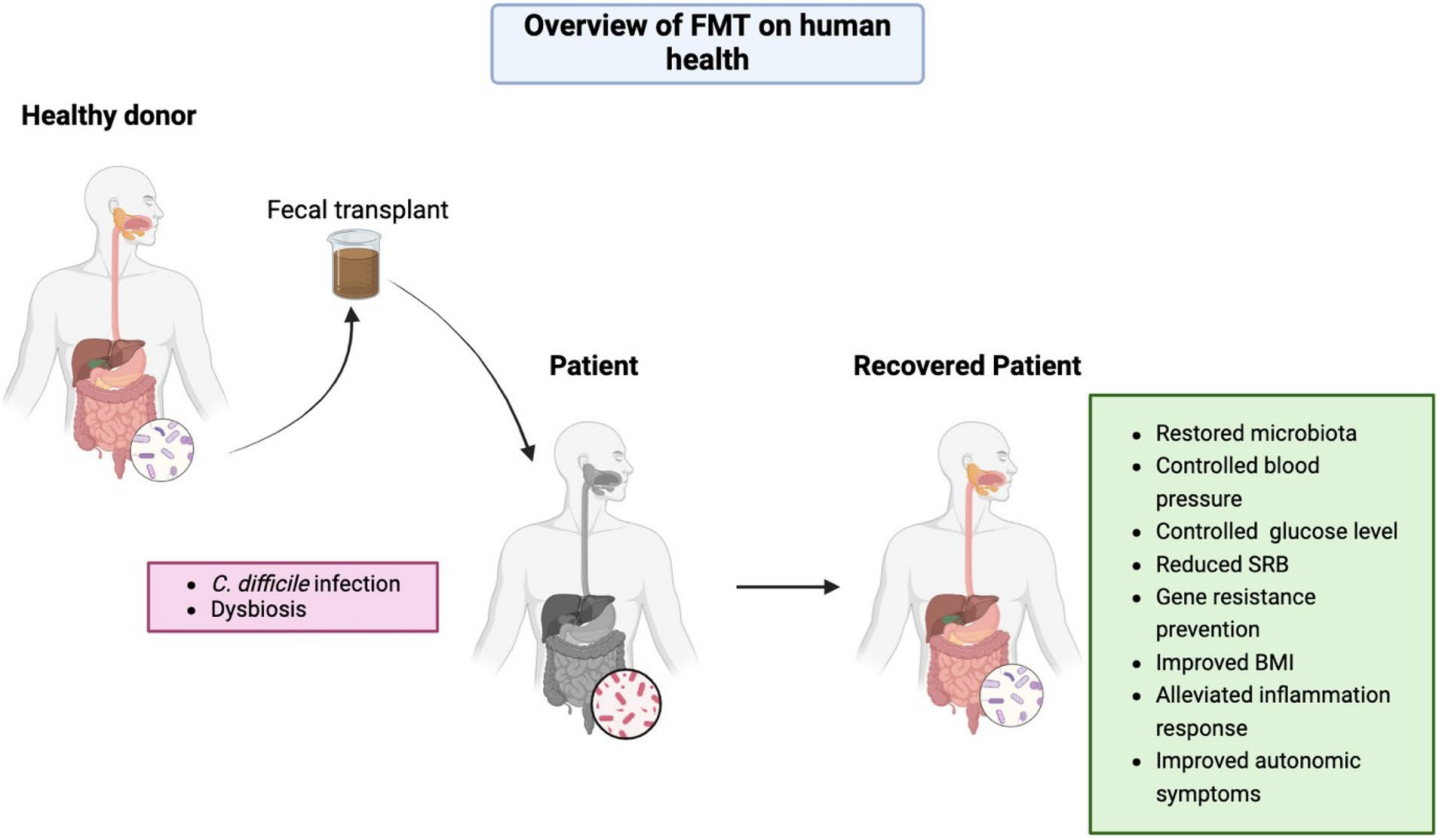
Overview of FMT effects on human health. Figure was created in https://BioRender.com.

Although FMT is generally well-tolerated, it dose carry risks requiring careful consideration. Transient gastrointestinal disturbances and rare cases of pathogen transmission underscore the need for rigorous donor screening protocols (15). Microbial engraftment patterns reveal complex dynamics—while bacterial transfer shows dose-dependent colonization, fungal taxa exhibit limited transmission persistence, suggesting distinct ecological establishment mechanisms (16). These findings highlight the importance of multimodal microbiota analysis in treatment optimization. To this end, this review searched for articles on several databases using the key terms ‘FMT and human health’ for the last 5 years to give a current summary of this nonpharmacological therapy. The aim of this review is to provide a comprehensive summary of this approach, while also revealing some limitations and prospects for further study (Figure 1).

## 2 FMT in metabolic disorders

FMT has shown significant efficacy in metabolic disorders. For instance, in patients with severe obesity, FMT improved metabolic responses by reducing the abundance of Prevotella and increasing the engraftment of donor-specific bacteria such as *Faecalibacillus intestinalis*, *Christensenellaceae* spp., and *Roseburia* spp. (17). Moreover, FMT demonstrated clinical efficacy in patients with non-alcoholic fatty liver disease (NAFLD), with lean NAFLD patients responding better than obese NAFLD patients (18). In patients with type 2 diabetes mellitus (T2DM), FMT improved body mass index, insulin resistance, and GM, with *Bifidobacterium adolescentis*, *Chlorobium phaeovibrioides*, and *Synechococcus* sp. WH8103 significantly associated with clinical parameters of T2DM (19). FMT also reduced HbA1c%, blood glucose, and uric acid levels while increasing postprandial C-peptide levels, despite individual variability. The abundance of families *Rikenellaceae* and *Ruminococcaceae* in the fecal of patients may serve as potential biomarkers for selecting T2DM patients to receive FMT (20). In type 1 diabetes mellitus patients, FMT was associated with the preservation of residual *β* cell function, particularly linked to the abundance of *Prevotella* (21).

In addition to reversing symptoms, FMT is safe for patients with severe alcoholic hepatitis with reduced pathogenic taxa, such as *Campylobacter* and anaerobes (*Weisella*, *Parcubacteria* and *Leuconostocaceae*) and increased *Alphaproteobacteria* and *Thaumarcheota* (22). Furthermore, the safety of oral FMT in treating chronic kidney disease was validated, with improvement in renal function based on urea nitrogen and serum creatinine levels. This was exacerbated with *Firmicutes* and *Actinobacteria* abundance and the reduction of *Bacteroidetes* and *Proteobacteria* (23). Autologous-fecal-microbiota-transplantation (aFMT) also showed potential in weight control and metabolic improvement, especially when combined with a plant based or high-polyphenol diet, optimizing weight loss and glycemic control (24–26).

## 3 FMT in intestinal diseases

The safety and efficacy of FMT in treating intestinal diseases have been widely validated. For instance, FMT demonstrated significant efficacy in CDI (27). Specifically, the increase in alpha diversity and the abundance of Ruminococcaceae and Lachnospiraceae, alongside the reduction of Enterobacteriaceae, have been identified as key biomarkers for the successful eradication of CDI through FMT (28). Moreover, FMT formulations such as RBX2660 and RBX7455 showed durability and safety in preventing CDI recurrence (29, 30). Additionally, FMT has shown promise in treating irritable bowel syndrome (IBS) and inflammatory bowel diseases. For example, in patients with moderate to severe IBS, FMT not only improved quality of life but also reduced fatigue and abdominal symptoms (31). Furthermore, with increasing SCFAs in IBS patients following FMT administration, an inverse correlation between butyric acid levels and IBS symptoms was observed (32). Notably, FMT reversed IBS symptoms by increasing the abundance of *Akkermansia* and *Neisseria* and reducing *Desulfovibrio* and *Delftia* (33). Although the mechanisms underlying the crosstalk between GM and colonic enteroendocrine cells remain elusive, FMT has proven efficacy in enhancing the density of colonic enteroendocrine cells in patients with IBS (34).

In addition, specific bacterial and metabolite associations have been established following the administration of capsulized FMT in patients with ulcerative colitis (UC) (35). Similarly, a single fresh FMT induced the expansion of Bacteroidetes and reduction of Proteobacteria in patients with recurrent UC (36). Moreover, serum IL-6, IL-10, and TNF-*α* modification is predicted to be related to FMT efficacy in UC (37). Finally, FMT has emerged as a promising treatment for tyrosine kinase inhibitors-induced diarrhea and small intestinal bacterial overgrowth, with no reported adverse effects (38, 39).

## 4 FMT on behavioral changes and immune response

Emerging evidence highlights the potential of FMT in modulating behavioral changes via gut-liver-brain axis. For instance, FMT reduced alcohol preference and consumption in patients with alcohol use disorder by increasing the diversity of SCFAs and *Ruminococcaceae* (40). Additionally, with *Bacteroidetes* and *Firmicutes* dominance, FMT can alleviate anxiety and depression while restoring intestinal microecology in patients with irritable bowel syndrome with predominant diarrhea (41). Notably, combining FMT with human intestinal fluid transplantation in capsule form has shown promise in improving childhood autism behavior, with observable benefits emerging as early as the first month of treatment. These findings underscore the potential of this combinatorial approach as an innovative therapy for autism (42).

Beyond neurological effects, FMT demonstrates immunomodulatory properties. In psoriatic arthritis, FMT initiates a distinct immunological plasma protein signature characterized by elevated IFN-*γ* levels, suggesting systemic immune reprogramming (43). Similarly, for immune-mediated dry eye in Sjögren’s syndrome patients, FMT induces a donor-like microbiota profile that correlates with improved dry eye symptoms and balanced regulatory/effector T-cell dynamics (44). Furthermore, daily encapsulated oral FMT is associated with mucosal-associated invariant T cell cytokine production with clinical significance (45). Importantly, FMT also exhibits therapeutic efficacy in systemic lupus erythematosus, rheumatic diseases, and acute and chronic graft-versus-host disease (46–48).

## 5 FMT and antimicrobial resistance

FMT with *Firmicutes* spores is a potentially novel strategy for controlling the growth of pathogens, thereby reducing the risk of antimicrobial resistance gene (ARG) colonization in the gastrointestinal tract (49). The abundance of ARGs is significantly reduced by FMT in patients with cirrhosis (50). Furthermore, an overall bimodal pattern with reduced ARG transfer for one-week post FMT, which is typical of healthy donor commensal microbiota, has been observed in patients undergoing allogeneic hematopoietic cell transplantation (51). However, stable communities with resistance to ARGs have also been observed in their patients, indicating that the length of clinical trials is a factor. Similarly, among renal transplant recipients, it has been suggested that strain competition induced by FMT reduces the number of multidrug-resistant organisms (52).

## 6 FMT co-supplementation on human health

The combination of FMT with supplements such as selenium showed inhibitory effects on the occurrence of colorectal cancer by increasing the abundance of beneficial bacteria, regulating phenotype and metabolic pathways (53). Although this is an animal study, the combination of FMT and selenium provides a new approach for treating colorectal cancer. Additionally, co-administration of FMT with an anti-inflammatory diet induced remission in patients with mild-to-moderate UC over 1 year (54). Furthermore, lifestyle interventions combined with FMT also improved liver stiffness and lipid profile in patients with T2DM by expanding *Bifidobacterium* and *Lactobacillus* (55). Similarly, patients with metabolic syndromes, such as obesity, showed improved insulin sensitivity upon daily administration of low-fermentable fiber combined with FMT supplementation (56). However, despite reduced small intestinal permeability, insulin resistance was not affected by FMT (57) (Table 1).

**Table 1:** Summary of FMT medical interventions.

Theme	Disease	Key findings	References
FMT in metabolic disorders	Severe Obesity	FMT reduces *Prevotella* and enhances colonization of *Faecalibacillus intestinalis*, *Christensenellaceae* spp., and *Roseburia* spp.	(17)
	NAFLD	FMT attenuates fatty liver disease by reducing fat accumulation and improving GM dysbiosis. Lean NAFLD patients exhibit better GM reconstruction compared to obese patients. FMT modulates specific taxa, including reduced *Actinobacteria* and increased *Selenomonadales* and *Prevotella 2*.	(18)
	T2DM	FMT improves body mass index, insulin resistance, and GM. FMT reduces HbA1c%, blood glucose, and uric acid levels while increasing postprandial C-peptide levels. Abundance of *Bifidobacterium adolescentis*, *Bifidibacterium Pegasus*, and *Synechococcu* ssp.WH 8103 is significantly associated with T2DM clinical parameters.	(19)
	Type 1 Diabetes	FMT preserves endogenous insulin production in recently diagnosed patients. Small intestinal *Prevotella* was inversely related to residual β cell function *Desulfovibrio piger* and specific gene expressions predict preserved *β*-cell function post-FMT.	(21)
	Severe Alcoholic Hepatitis	FMT improves 90-day survival and reduces infections by modulating microbial communities. FMT reduces *Campylobacter* and *Leuconostocaceae*, *Weisella*,while increasing *Alphaproteobacteria* and *Thaumarcheota*.	(22)
	Chronic Kidney Disease	FMT enhances renal function (serum creatinine and urea nitrogen) FMT modulates microbiota by reducing *Firmicutes* and *Actinobacteria* while increasing *Bacteroidetes* and *Proteobacteria*.	(23)
FMT in intestinal diseases	CDI	FMT had increased microbial *α* diversity, enriches *Ruminococcaceae* and *Lachnospiraceae*, depletes *Enterobacteriaceae*. RBX2660 is safe and effective in reducing CDI recurrence by reguLating GM. RBX7455 is safe and effective at preventing recurrent CDI by increasing *Bacteroidia* and *Clostridia.*	(28, 29, 30)
	IBS	FMT improves quality of life, reduces fatigue and abdominal symptoms, and increases SCFAs. FMT modulates GM by increasing *Akkermansia* and *Neisseria* while reducing *Desulfovibrio* and *Delftia*.	(31, 33)
	UC	FMT alleviates symptoms by increasing *Bacteroidetes* and reducing *Proteobacteria*. Changes in serum IL-6, IL-10, and TNF-α are associated with FMT efficacy.	(36, 37)
FMT on behavioral changes and immune response	Alcohol Use Disorder	FMT reduces alcohol preference and consumption. FMT increases SCFAs and *Ruminococcaceae* diversity.	(40)
	Anxiety and Depression	FMT alleviates anxiety and depression in IBS patients. FMT increases *Bacteroidetes* and *Firmicutes.*	(41)
	Childhood Autism	FMT combined with human intestinal fluid transplantation improves autistic behaviors within 1 month.	(42)
	Psoriatic Arthritis	FMT significantly alters levels of 12 proteins, including sustained elevation of IFN-*γ*.	(43)
	Sjögren’s Syndrome	FMT induces donor-like microbiota distribution, improves dry eye symptoms, and balances T-cell dynamics. FMT modulates genera such as *Faecalibacterium*, *Prevotella*, and *Alistipes*.	(44)
	Systemic Lupus Erythematosus	FMT enriches SCFAs-producing taxa, reduces inflammation-related taxa, and decreases IL-6 levels. Specific microbiota signatures are associated with SRI-4 response.	(47)
	Graft-Versus-Host Disease	FMT induces complete clinical response within one month. FMT increases α-diversity, and enriches butyrate-producing bacteria (*Clostridiales* and *Blautia*).	(87)
FMT and antimicrobial resistance	Liver Cirrhosis	FMT reduces antimicrobial resistance genes (ARGs) and increases duodenal mucosal diversity. FMT modulates taxa such as *Ruminococcaceae* and *Bifidobacteriaceae*.	(50, 88)
	Kidney Transplant Recipients	FMT reduces multidrug-resistant microorganisms through strain competition. Key taxa (e.g., *Akkermansia muciniphila*, *Faecalibacterium prausnitzii*, *Alistipes putredinis*, *Phocaeicola dorei*, *Phascolarctobacterium faecium*, *Alistipes species*, *Mesosutterella massiliensis*, and *Barnesiella intestinihominis*) from a single donor successfully engrafted in recipients.	(52)
FMT co-supplementation on human health	UC	FMT combined with an anti-inflammatory diet induces remission in mild-to-moderate UC patients.	(54)
	Obese patients with T2DM	Repeated FMTs enhance microbiota engraftment and improve liver stiffness and lipid profiles. Combining FMT with lifestyle intervention increases *Bifidobacterium* and *Lactobacillus*.	(55)
	Metabolic Syndrome	FMT combined with low-fermentable fiber improves insulin sensitivity.	(56)

## 7 Limitations and safety of FMT

While FMT standardization continues to advance in clinical practice, critical challenges persist in optimizing its therapeutic applications. Three primary safety concerns warrant attention. First, procedure-related complications associated with administration methods, as evidenced by gastroduodenoscopic risks observed with commercial anaerobic-cultivated products despite their gastrointestinal symptom relief potential (58). Second, the dual-edged nature of immune modulation emerges when donor-derived antigens paradoxically activate immune cells, potentially exacerbating pre-existing conditions—a phenomenon underscoring the need for precision-engineered microbial consortia targeting specific pathologies like intestinal barrier dysfunction (59). Finally, preclinical models reveal systemic consequences through FMT-induced elevations in central/peripheral inflammatory mediators coupled with compromised intestinal mucosal integrity, mechanistically linking gut microbiota alterations to anxiety-depressive manifestations (60).

The therapeutic landscape of FMT demonstrates striking condition-specific variability. In gastrointestinal disorders, while showing efficacy in ameliorating certain IBS symptoms, it fails to normalize stool frequency or resolve abdominal pain (61). Metabolically, its actions exhibit paradoxical selectivity: Although reducing visceral adiposity in obese adolescents, FMT does not translate to clinically meaningful weight reduction (62). This dichotomy extends to molecular mechanisms, where epigenetic reprogramming of immune cells and plasma metabolome modifications correlate with improved insulin sensitivity (63), yet cachexia progression remains unaffected in metastatic cancer models (64).

Emerging safety data necessitate judicious clinical translation. Of particular concern, combination therapy trials incorporating UC exclusion diets were terminated prematurely despite initial mucosal healing observations, highlighting unforeseen risks in therapeutic synergies (65). Complementing these clinical findings, preclinical evidence demonstrates a gut-brain axis disruption mechanism: FMT-induced intestinal hyperpermeability not only elevates systemic inflammation biomarkers but also precipitates anxiety-depressive phenotypes (60). While demonstrating promise in managing steroid-refractory gastrointestinal graft-versus-host disease (66), these collective findings underscore the imperative for longitudinal safety surveillance and mechanistic investigation into microbiota-host interactions.

## 8 Prospects and recommendations for FMT

Within the first 7 days of FMT administration, donor-derived species, SCFAs, and tryptophan metabolites expanded despite a low degree of donor-recipient microbiome similarity (67). Importantly, individual species or strain-driven metabolite changes in the lower gastrointestinal tract of patients with acute graft-versus-host disease highlight the need for careful donor selection. Given that donors are required to have an abundance of selective taxa, two critical factors emerge: variation and stability. For example, multi-donor FMT may alter gut microbiota function due to microbiome competition, thus underscoring the importance of selecting donor FMTs that dominate strain engraftment. Such selection should prioritize diversity, as engrafted strains can induce enterotype-level shifts that alter metabolic potential (68). Furthermore, the abundance of the keystone genera, *Prevotella*, *Faecalibacterium*, and *Bacteroides* and increased microbial diversity are biomarkers of FMT capsule administered to treat IBS (69).

To optimize FMT efficacy, previous fecal microbiome profiles should be used as biomarkers during patient recruitment, ensuring unbiased selection (70). Moreover, FMT regimens must demonstrate effectiveness even for rare diseases under trial, such as chronic pouchitis (71). As the delivery methods for FMT may have distinct effects, there is a need to establish the method with the most effective treatment (72). Additionally, while FMT has shown acceptability, safety, and feasibility in treating major depressive disorder, a comprehensive evaluation of its clinical efficacy based on standardized protocols is still required (73). Similarly, although FMT capsules alter both serum metabolomics and gut microbiota in mild cognitive impairment, this does not confirm their safety or efficacy (74).

The effectiveness and safety of FMT are significantly influenced by disease severity and dosage composition (75). For instance, single-dose FMT is not recommended for maintaining remission in UC, despite its clinical efficacy in microbiome engraftment (76, 77). In contrast, oral FMT emerges as a feasible option for UC treatment. Specifically, Microbial Ecosystem Therapeutic 2, an oral encapsulated formulation comprising 40 lyophilized bacterial species, has demonstrated safety and efficacy in UC, even in children with cytomegalovirus infection (78, 79). However, antibiotic pretreatment during FMT poses a significant limitation in clinical trials, highlighting the need for alternative approaches (80). One such alternative is washed microbiota transplantation, which appears to be both effective and safe, despite the lack of clinical significance in delivery methods (81, 82).

To further refine FMT applications, donor-recipient species genome bins can predict strain transfer dynamics and specific microbial interactions, thereby improving metabolic health (83). Additionally, donor age and sex play critical roles in FMT success, as strain-level variations may influence outcomes in a species-specific manner (84). While both sexes respond similarly to FMT in IBS patients, females tend to exhibit increased fecal *Alistipes*, regardless of administration method (85). Nevertheless, conflicting results from IBS studies suggest that sex may not be a determining factor in FMT outcomes, indicating the need for further investigation (86).

## 9 Conclusion

FMT shows therapeutic promise, particularly in reversing diseases and disorders. In addition, positive results in animal models suggest its potential for human clinical trials; however, safety and efficacy must be rigorously monitored before clinical adoption. While the FDA has classified FMT as a “drug” due to its ability to treat and prevent disease, emerging adverse effects highlight the need for further large cohort studies and long-term monitoring to establish stable benefits. In addition, several challenges remain, including pediatric use, regimen optimization, and ensuring patient confidence in safety and efficacy. In addition, donor screening is essential to prevent disease transmission, and stool variability poses significant regulatory challenges for the FDA. Looking ahead, encapsulated stool formulations represent an important future direction, offering non-invasive oral delivery that could increase patient acceptance and broaden clinical applications.
